# Oncogenic viral protein HPV E7 up-regulates the SIRT1 longevity protein in human cervical cancer cells

**DOI:** 10.18632/aging.100028

**Published:** 2009-03-02

**Authors:** Simon J. Allison, Ming Jiang, Jo Milner

**Affiliations:** Yorkshire Cancer Research P53 Research Unit, Department of Biology, University of York,; York, YO10 5DD, UK; ^1^current address: Cancer Research UK, Lincoln's Inn Fields Laboratories, London, WC2A 3PX, UK

**Keywords:** HPV E7; HPV E6; SIRT1; p53; aging; cancer

## Abstract

Senescence is blocked in human cervical keratinocytes infected with high risk human papillomavirus (e.g. HPV type16). Viral oncoproteins HPV E6 and HPV E7 access the cell cycle via cellular p53 and retinoblastoma proteins respectively. Previously we have shown that HPV E7, not HPV E6, is also responsible for cervical cancer cell survival (SiHa cells; HPV type16). We now present evidence that SIRT1, an aging-related NAD-dependent deacetylase, mediates HPV E7 survival function in SiHa cervical cancer cells. Moreover, HPV E7 up-regulates SIRT1 protein when expressed in primary human keratinocytes. Conversely, SIRT1 levels decrease following RNAi-mediated silencing of HPV E7 in SiHa cells. Silencing HPV E6 has no effect on SIRT1 but, as expected, causes marked accumulation of p53 protein accompanied by p53-mediated up-regulation of p21. However, p53 acetylation (K382Ac) was barely detectable. Since p53 is a known SIRT1 substrate we propose that elevated SIRT1 levels (induced by HPV E7) attenuate p53 pro-apoptotic capacity via its de-acetylation. Our discovery that HPV E7 up-regulates SIRT1 links a clinically important oncogenic virus with the multi-functional SIRT1 protein. This link may open the way for a more in-depth understanding of the process of HPV-induced malignant transformation and also of the inter-relationships between aging and cancer.

## Introduction

The processes of aging and cancer share many characteristics despite their opposing phenotypes of senescence versus immortalisation [1]. In both cases for example there is accumulation of DNA damage and the loss of genomic integrity [1,2]. Two major players which impinge upon chromatin structure and the maintenance of genomic integrity in mammalian systems are p53 [3-6] and SIRT1 [7-9]. Reciprocal regulation occurs between SIRT1 and p53 in which SIRT1 binds and de-acetylates activated p53 [10-12] whilst activated p53 down-regulates SIRT1 translation via miR-34a  [13].  The p53 protein is best known as  a tumour suppressor but is becoming increasingly recognized as a factor also involved in senescence and aging [14,15]. Conversely SIRT1, one of seven mammalian sirtuins and an NAD-dependent de-acetylase [16], first emerged as a potential anti-aging factor (reviewed in [17,18]) but is now also implicated in a number of age-related disease processes including tumour development (which it suppresses) [9,19] and cancer cell survival (which it supports) [20].

The yeast model provided the initial evidence linking SIRT1 with aging. Thus Sir2, the ancestral homologue of SIRT1, is a yeast NAD-dependent deacetylase and longevity factor [16,17]. In budding yeast Sir2-mediated silencing at rDNA loci is crucial for chromatin compaction and suppresses homologous recombination [16]. In this way Sir2 suppresses the formation and accumulation of extra-chromosomal rDNA circles, one of the primary causes of yeast aging [21]. Sir2 thus functions as a longevity factor in yeast in which it also silences mating type loci, is responsive to growth conditions and calorie restriction [16]. Recent evidence now indicates that, in mammals, its homologue SIRT1 also functions as a longevity factor [8,17,22-24] and is responsive to diverse stresses many of which also activate the p53 tumour suppressor [8,17,25,26].

Much of our understanding of the molecular biology of cancer derives from early studies in which DNA tumour viruses were employed and the mechanisms by which they induce cell transformation from normal to cancerous growth were elucidated. Indeed studies with DNA tumour viruses led to the initial discovery of p53, the major tumour suppressor in humans [27]. One of these viruses was the human papillomavirus (HPV) which targets p53 via the HPV E6 protein. High risk HPV types are now recognised as the cause of human cervical cancer (see for example [28]) and responsible for some 500,000 newly diagnosed cases worldwide each year. High risk HPV types include HPV16 and HPV18 [28]. Although vaccination strategies are now employed to protect uninfected children, there remain millions of HPV-infected women who are at risk of developing cervical cancer. The switch from HPV latency to malignancy is poorly understood. This transition appears to involve integration of the viral episome into the host cell genome together with enhanced expression of two viral oncoproteins HPV E6 and HPV E7 [29]. Presumably some event favours stable integration of viral chromatin into host chromatin. Understanding this crucial switch may enable the development of novel therapies designed to protect HPV-positive patients from progression to malignancy.

HPV E6 and E7 are expressed as bicistronic mRNA. In this current work we have exploited a previous unexpected observation, namely that in HPV16-positive SiHa cells it is possible to selectively and individually silence E6 and E7 by RNA interference (RNAi) despite the contiguous nature of their mRNAs [30]. Here we have confirmed and extended this important observation, and studied the individual effects of HPV E6 and E7 expression in human cervical cancer cells (SiHa) and in primary human epithelial keratinocytes (the cell type infected by HPV *in vivo*).

It is already established that HPV16 E6 targets the p53 protein for rapid degradation with consequential loss of p53 tumour suppressor functions, including maintenance of chromosomal integrity. We have now discovered that the second HPV viral oncogene, HPV16 E7, targets SIRT1. Specifically we demonstrate (i) that exogenous expression of HPV E7 (but not HPV E6) in primary human keratinocytes induces abnormally high levels of the SIRT1 protein, similar to those observed in human cervical cancer cells, and (ii) that HPV E7 (but not HPV E6) is required to maintain the abnormally high levels of SIRT1 protein expressed in cervical cancer cells. The ability of HPV E7 to up-regulate SIRT1 appears to be linked with HPV E7-mediated suppression of apoptosis in cervical cancer cells [30] since our current work also demonstrates that SIRT1 suppresses apoptosis in SiHa cells. In addition to up-regulating SIRT1 we show that HPV E7 induces global site-specific histone H3 modifications and, in synergy with the kinase aurora B, up-regulates the anti-apoptotic survivin protein. These various changes are predicted to affect chromatin structure and to promote cell survival.

Our discovery that HPV E7 influences the expression of SIRT1 provides the first link between an oncogenic virus and the aging-related SIRT1 protein, and enables access to diverse SIRT1-dependent cellular regulatory systems. This link may open the way for a more in-depth understanding of the inter-relationships between aging and cancer. It may also provide insight into the mechanism of chromosomal re-distribution during the switch from latent to malignant HPV viral infection, and also for the maintenance of HPV-induced malignancy.

## Results and discussion

### Individual knock-down of HPV16 E6 and E7 in SiHa cells

**Figure 1.**
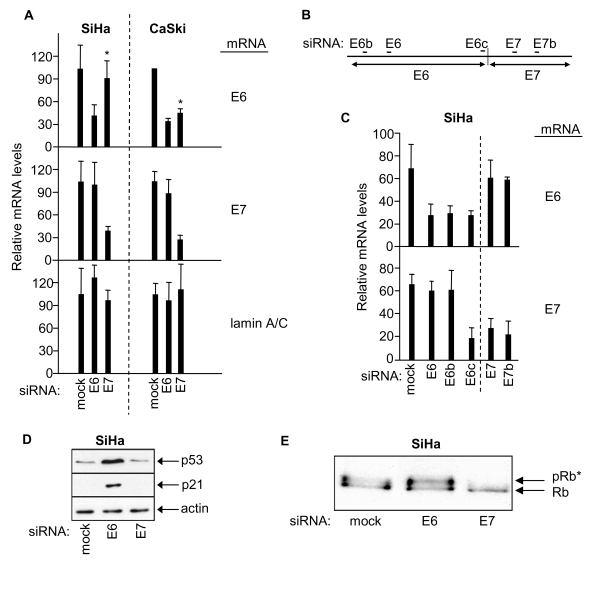
RNAi-mediated knock-down of HPV E6 and HPV E7 in SiHa and CaSki cells, and effects on p53 and retinoblastoma protein. (**A**) mRNA qRT-PCR determinations 48h post-transfection as indicated, mean ± s.d. of three determinations. Asterix indicates differential effect of E7 siRNA on E6 mRNA levels in SiHa versus CaSki cells. (**B**) Relative positions of siRNA sequences along the bicistronic E6/E7 transcript. (**C**) Relative levels of E6 and E7 mRNAs 48h post-transfection of SiHa cells with the indicated siRNAs. (**D, E**) Immunoblots showing effects of E6 or E7 depletion on levels of p53, p21 and hyperphosphorylated Rb (pRb*) in SiHa cells.

For this study we exploited the ability of RNAi to separately knockdown the viral oncogenes HPV16 E6 and HPV16 E7 in HPV16-positive SiHa cervical cancer cells. Individual knockdown of HPV E6 and E7 by their respective siRNAs was verified by quantitative RT-PCR (Figure 1A, left panel), thus confirming our previous observations [30].  This effect appears peculiar to SiHa cells since similar individual knock-down of E6 and E7 was not obtained for HPV16-positive CaSki cervical cancer cells in which E7 siRNA induced knock-down of both HPV E6 and E7 expression (Figure 1A, right panel, asterix).

The mechanistic basis for the differential specificity of E7 siRNA in SiHa versus CaSki cells is at present unclear. However, it is noteworthy that the bicistronic E6/E7 transcripts produced in SiHa and CaSki cells differ in their junctional organisation [29,31] and in their predicted secondary structures [29]. Secondary structure could affect accessibility and/or progression of RNAi-associated machinery along the bicistronic transcript. Thus it may be that an E6/E7 mRNA boundary effect is imposed by RNA secondary structure which segregates E6 and E7 RNAi-mediated silencing of the bicistronic transcript in SiHa cells.

RNAi-mediated knockdown of HPV E6 and E7 in SiHa cells was further investigated using additional siRNAs (Figure 1B). Selective knockdown of E7 mRNA was obtained without effect on E6 mRNA levels using two independent E7 siRNAs (E7 and E7b siRNAs, Figure 1B and 1C). Conversely, two of three E6 siRNAs selectively targeted E6 mRNA for degradation without effect on E7 mRNA (E6 and E6b siRNAs, Figure 1B and 1C). Interestingly, a third E6 siRNA (E6c), designed to target the junction region of the E6/E7 bi-cistronic transcript, induced knock down of both E6 and E7 mRNA sequences (Figure 1B and 1C). Overall these results support the concept of a boundary effect influencing RNAi-mediated degradation at the HPV E6/E7 mRNA junction in SiHa cells.

HPV E6 is known to target p53 for degradation and, as expected, E6 knockdown resulted in elevated p53 protein levels and up-regulation of p53-dependent p21 expression (Figure 1D; see also [30]) but had no effect on the phosphorylation status of the retinoblastoma protein (Rb; Figure 1E). HPV E7 on the other hand is known to induce hyperphosphorylation of Rb and, as expected, depletion of E7 via E7 siRNA resulted in loss of hyper-phosphorylated Rb (Rb*, Figure 1E; see also [30]) but was without effect upon p53 protein levels or p21 expression (Figure 1D). These combined observations are absolutely consistent with selective and individual RNAi-mediated knock-down of HPV E6 and E7 in human cervical cancer SiHa cells.

### E7 silencing has site-specific effects on global histone H3 modifications

**Figure 2.**
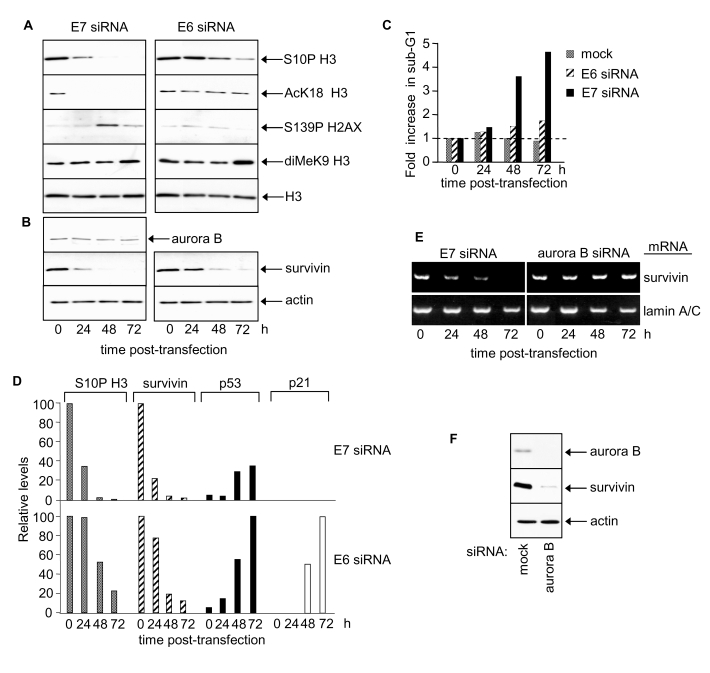
HPV E7 maintains survivin protein and induces site-specific changes in histone H3 modifications. (**A**) Histone H3 modifications at 24, 48 and 72 h post-transfection with E6 or E7 siRNA. Equivalent exposures for each pair of immunoblots shown. (**B**) Aurora B and survivin protein levels following E6 or E7 silencing. (**C**) Cell death after E6 or E7 siRNA treatment. (**D**) Relative changes in S10P H3, survivin, p53 and p21 proteins in response to E7 or E6 siRNA. (**E**) RT-PCR determinations of survivin and lamin A/C mRNA levels in SiHa cells as indicated. (**F**) Immunoblots showing effect of aurora B silencing on survivin protein levels at 48h post siRNA transfection.

We next examined the epigenetic effects of HPV E6 and E7 upon global modifications of histone H3. Such modifications represent prime targets for deregulation in cancer and/or aging [4,5,32,33]. Depleted levels of phosphorylated S10 of histone H3 by ~3 fold were observed 24h following transfection with E7 siRNA and by 48h histone H3 S10P was barely detectable (Figure 2A, left panel and 2D). Thus HPV16 E7 appears, directly or indirectly, to increase global histone H3 S10 phosphorylation. In contrast, levels of S10P H3 were unchanged 24h following transfection with E6 siRNA (Figure 2A, right panel and 2D). However, a decrease was observed by 48h and 72h and inversely correlated with increasing p53 levels (Figure 2D). Since p53 decreases global histone H3 S10P [4,34] the observed decrease in histone H3 S10P following HPV E6 RNAi is likely to be attributable to p53 induction. Levels of acetylated K18 of histone H3 also fell in response to E7 silencing (Figure 2A, left panel) but did not change in response to E6 silencing (Figure 2A, right panel). Thus histone H3 K18 acetylation levels are selectively up-regulated by HPV E7. The changes in S10P and K18Ac of histone H3 precede apoptosis in HPV E7-depleted cells (Figure 2A, Figure 2C for sub-G1 content; see also [30]). Phosphorylation of histone H2AX at S139 is associated with apoptotic events [35] and levels peaked transiently 48h post-transfection with E7 siRNA (Figure 2A) co-incident with the onset of apoptosis ([30]; Figure 2C and data not shown). There was no change in histone H2AX following RNAi-mediated silencing of HPV E6 (Figure 2A).

Acetylated K9 and K14 histone H3 levels were very low in SiHa cells and no change was detected following transfection with either E6 or E7 siRNA (data not shown). Treatment with trichostatin A, a class I and II histone deacetylase inhibitor, induced a dramatic increase in both K9Ac and K14Ac of histone H3 (data not shown) indicating high deacetylase activity at these sites in SiHa cells. Neither E6 nor E7 significantly affected K9 methylation of histone H3 in SiHa cells (Figure 2A).

### HPV E7 maintains elevated survivin levels in cervical cancer cells

To explore how E7 silencing might decrease S10P we examined levels of aurora B and survivin. Aurora B is the principal histone H3 S10 kinase in human cells [36] and its activity is regulated by survivin [37]. Aurora B protein levels did not change in response to E7 silencing (Figure 2B). Interestingly, however, E7 silencing depleted survivin protein levels ~5 fold by 24h post-transfection (Figure 2B and 2D), with little, if any, detectable protein by 48 and 72h.  These results identify survivin as a cellular target of HPV16 E7 and suggest that E7 can, directly or indirectly, upregulate survivin expression and/or stabilise survivin protein.

In the mouse model for BRCA1 tumor suppressor function there is evidence that wild type BRCA1 suppresses survivin expression via SIRT1-dependent epigenetic modification of histone H3 [38]. Our own future studies will determine if, in human cervical cancer, HPV16 E7 impacts upon survivin expression via a SIRT1-dependent mechanism. This will be particularly interesting given our discovery that HPV E7 up-regulates SIRT1 protein levels in human cervical cancer SiHa cells (see below).

E6 silencing caused a delayed reduction in survivin protein levels (Figure 2B and 2D). The decrease in survivin in response to E6 silencing was inversely correlated with p53 protein levels, suggesting that this effect is mediated by p53 (Figure 2D) [39-41]. Lamin A/C silencing, a negative control, had no effect on survivin protein levels (data not shown).

### HPV E7 up-regulates survivin mRNA whilst aurora B sustains survivin protein levels

HPV E7 silencing also resulted in reduced mRNA levels of survivin (Figure 2E) indicating that HPV E7 increases host cell survivin transcription and/or mRNA half-life. Interestingly, although aurora B had no effect on survivin mRNA levels (Figure 2E) it appeared to sustain survivin protein levels (Figure 2F). It is possible that phosphorylation by aurora B [37,42] stabilises survivin protein.

### Growth effects of survivin and aurora B in SiHa cells

**Figure 3.**
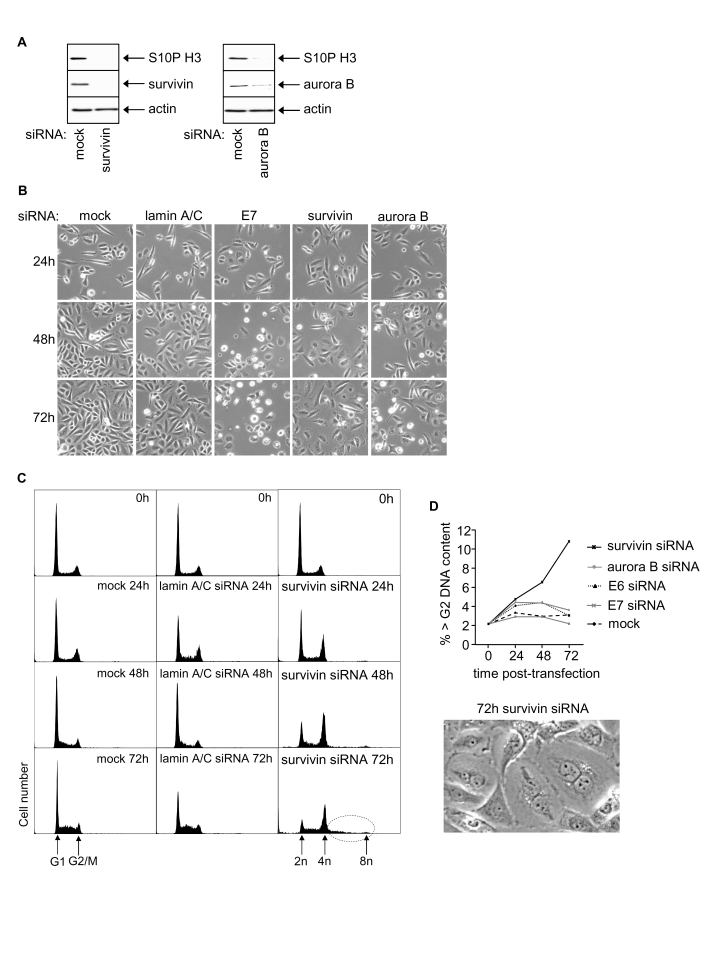
Effects of RNAi-mediated silencing of survivin and aurora B on S10P histone H3 and SiHa cell phenotype. (**A**) Survivin, aurora B and S10P histone H3 protein levels 48h post-transfection. (**B**) Phase contrast images of SiHa cells as indicated. (**C**) Cell cycle distribution at 0, 24, 48 and 72h post-transfection with indicated siRNAs. (**D**) Induction of polyploidy in SiHa cells following  survivin depletion indicated by percentage of >G_2_ cells and appearance of multinuclear cells.

Survivin knockdown in SiHa cells resulted in reduced cell growth but failed to induce apoptosis (Figure 3A, 3B; data not shown). Progressive reduction in G_1_ cells from ~60% to ~11% was observed 72h post-transfection (Figure 3C, quantitated by histogram deconvolution, see Methods) and cells accumulated in G_2_/M. There was also a significant increase in the proportion of cells with >G_2_/M DNA content (Figure 3C, dotted circle; and 3D), suggesting that a proportion of the G_2_/M cells are still cycling but had failed to undergo cytokinesis. Indeed, two or more nuclei were visible in many of the enlarged cells resulting from transfection with survivin siRNA (Figure 3D).

### HPV16 E7 up-regulates SIRT1 protein levels in cervical cancer cells

**Figure 4.**
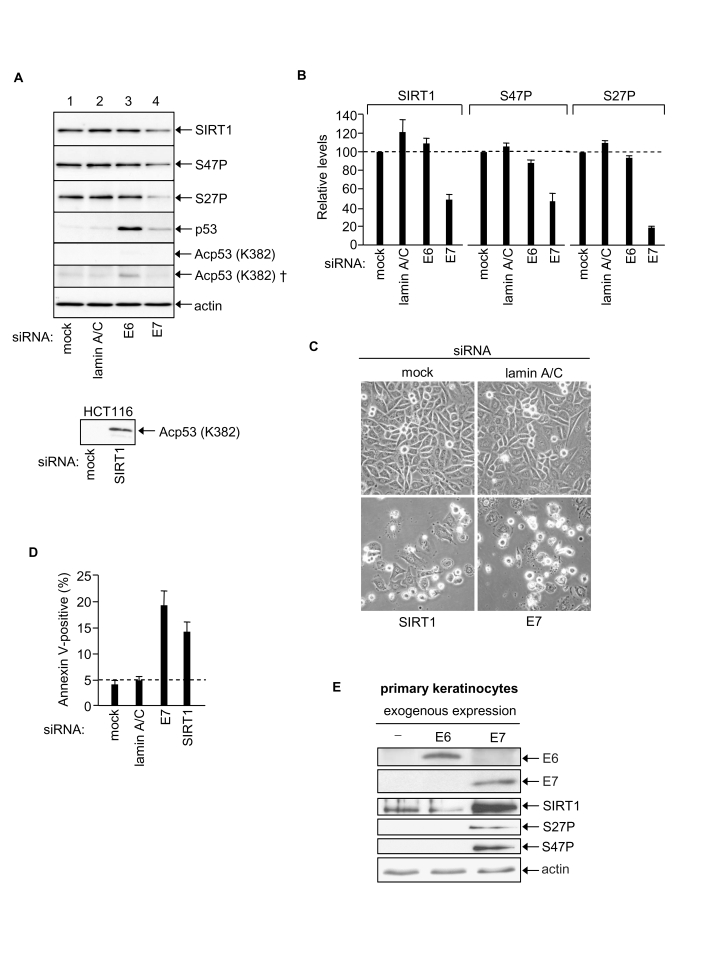
HPV E7 enables SiHa cervical cancer cell survival via up-regulation of SIRT1 protein levels. (**A**) Equal amounts of protein analysed by immunoblotting as indicated, upper panels SiHa cells (50 μg protein). Bottom panel HCT116 cell positive control for p53 K382Ac detection (40 μg protein, 2 minute exposure). Note that p53 K382Ac is undetectable in SiHa cells (5 min exposure) and requires 2 h exposure for detection (†). (**B**) Relative levels of SIRT1, SIRT1 S47P and SIRT1 S27P 48h post-transfection with indicated siRNAs, mean of two experiments. (**C**) Phase contrast images of SiHa cells post-transfection with the indicated siRNAs. (**D**) Apoptotic SiHa cells 48h post-transfection with the indicated siRNAs. (**E**) Primary human keratinocytes 48h post-transfection with expression vectors for HPV E6 and HPV16 E7 and equivalent samples immunoblotted for HPV E6, HPV E7, SIRT1, SIRT1 S27P and SIRT1 S47P.

Previously we have shown that SIRT1 acts as a cancer-specific survival factor in a range of epithelial cancer cell lines [20]. This raised the possibility that SIRT1 might mediate the anti-apoptotic effects of HPV16 E7 [30]. Moreover SIRT1 protein levels are abnormally elevated in a range of human epithelial cell lines, including SiHa cervical cancer cells [25]. We now demonstrate that RNAi-mediated silencing of HPV E7 in SiHa cells down-regulates SIRT1 protein levels by ~50% 48h post-transfection with E7 siRNA (Figure 4A and 4B). In these same cells the levels of the pro-apoptotic E3 ligase Itch and anti-apoptotic c-FLIP (c-FLIP_S_ and c-FLIP_L_) were unaffected by HPV E7 knock-down (data not shown) demonstrating that the reduction in SIRT1 protein levels reflects a selective effect of HPV E7 depletion. In contrast to HPV E7 depletion, the depletion of HPV E6 had no effect on levels of SIRT1 protein (Figure 4A and 4B).  We conclude that HPV E7, but not HPV E6, is required to maintain the abnormally high levels of SIRT1 protein characteristically observed in SiHa cervical cancer cells. Our results thus identify the longevity protein SIRT1 as a novel cellular target of the viral oncogene HPV16 E7 in human cervical cancer cells.

The expression of SIRT1 in mammalian cells is subject to multiple control mechanisms operating at the levels of transcription, transcript half-life, translation and protein half-life [13,25,26,43]. In the present study we found that depletion of HPV E7 in SiHa cells had no significant effect on SIRT1 mRNA levels (data not shown) suggesting that the effect of HPV E7 on SIRT1 protein expression levels is post-transcriptional. Recently, we have reported that SIRT1 protein stability in human colorectal cancer cells (HCT116) is dependent upon JNK2 and is linked with SIRT1 phosphorylation at S27 [25]. A similar effect may contribute to the enhanced level of SIRT1 in SiHa cervical cancer cells since HPV E7 silencing caused ~ 80% reduction in SIRT1 S27P, whereas levels of SIRT1 phosphorylation at a second site, S47, paralleled the decrease in total SIRT1 (Figure 4A and 4B).

### Depletion of SIRT1 in SiHa cells induces apoptosis

Depletion of SIRT1 in SiHa cells induced apoptosis (Figure 4C and 4D) demonstrating that cellular SIRT1 enables SiHa cervical cancer cell survival. This is consistent with our previous observation that SIRT1 functions as a cancer-specific survival factor in a range of human epithelial cancer cell lines [20]. Our present results indicate that HPV E7-dependent elevation of SIRT1 protein levels plays an essential pro-survival role in human cervical cancer cells. SIRT1-mediated suppression of apoptosis would thus explain the survival function of HPV E7 in SiHa cells (this work and Ref. 30). Moreover, since SIRT1 is an NAD-dependent de-acetylase with multiple cellular targets, the discovery that HPV E7 up-regulates SIRT1 protein levels identifies a new mechanism by which HPV can access diverse SIRT1-dependent regulatory systems in human cervical keratinocytes.

### Exogenous expression of HPV E7 increases SIRT1 protein levels in primary human keratinocytes

The above results strongly indicate that HPV E7 in some way targets the cellular SIRT1 protein and maintains its expression at abnormally high levels in cervical cancer cells. The effect is specific for HPV E7 since no effects on SIRT1 were observed following RNAi-mediated silencing of HPV E6 under identical conditions (see above). The HPV E7 effect on SIRT1 could be a late event in the process of malignant cell transformation by the HPV virus. Alternatively HPV E7-mediated up-regulation of SIRT1 might play a causative role in malignant cell transformation. In favour of the latter alternative we show that SIRT1 suppresses apoptosis in SiHa human cervical cancer cells (SiHa; Figure 4C and 4D).

If HPV E7-mediated up-regulation of SIRT1 plays a causative role in malignant cell transformation we reasoned that this effect should be detected as an early event in the process of keratinocyte transformation. To test this primary human keratinocytes were exposed to high level HPV E7 expression: i.e. as occurs following integration of the HPV viral genome into host cell chromatin during malignant transformation *in vivo*. For this purpose we constructed individual expression vectors using HPV E6 and HPV E7 freshly cloned from the SiHa cells employed throughout this study (see Materials and Methods). Primary human keratinocytes were transfected with HPV E6 or HPV E7 expression vectors and exogenously expressed E6 and E7 proteins were detected by immunoblotting (Figure 4E). Samples were also probed for SIRT1 protein, SIRT1 S27P and SIRT1 S47P (in SiHa cells SIRT1 protein is phosphorylated at both S27 and S47; [25]).

The results clearly show that SIRT1 protein levels increase dramatically within 48h expression of exogenous HPV E7. We have previously noted lack of detectable SIRT1 S27 and S47 phosphorylation in primary human keratinocytes [25] and this is confirmed here (Figure 4E). However both S27P and S47P became detectable following exogenous expression of HPV E7. This may simply reflect a detection threshold or, alternatively, be mechanistically linked with SIRT1 protein accumulation (S27P correlates with SIRT1 protein half-life in HCT116 colorectal cancer cells) [25]. It is also possible that SIRT1 protein accumulation in response to HPV E7 involves its relocation within the cell, and/or novel protein-protein interactions, and/or other post-translational modifications such as sumoylation. Further studies are required to elucidate the mechanism of SIRT1 protein accumulation in response to HPV E7.

In contrast to HPV E7 the exogenous expression of HPV E6 failed to induce a change in SIRT1 protein level in primary human keratinocytes (Figure 4E; although p53 levels were depleted, data not shown). Thus increased SIRT1 protein expression levels induced by HPV E7 (i) cannot be attributable to a cellular stress response induced by expression of a foreign, viral protein, and (ii) cannot be induced by HPV E6.

We therefore conclude that HPV E7 selectively induces increased SIRT1 protein levels and that this occurs within 48h of exogenous E7 expression in primary human keratinocytes. Conversely, in SiHa cells naturally expressing HPV E7 via the integrated HPV viral genome, E7 silencing induces a decrease in SIRT1 protein. Thus our overall results indicate that HPV E7 positively regulates SIRT1 protein levels and that SIRT1 functions as a down-stream mediator of HPV E7 in sustaining malignant cell survival.

### A new model for cell transformation by HPV E6 and E7

**Figure 5.**
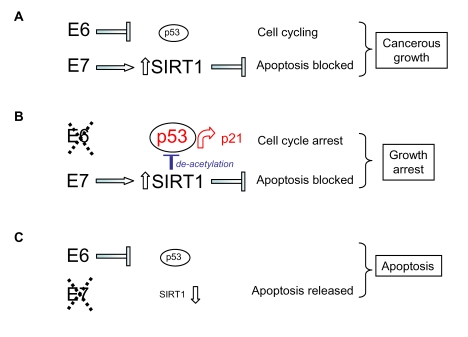
Model for the respective effects of HPV E6 and HPV E7 on human cervical cancer cell survival and proliferation taking into account (**A**) up-regulation of SIRT1 protein by HPV E7, (**B**) SIRT1-mediated de-acetylation of p53 and (**C**) SIRT1 cervical cancer cell survival functions (see text).

Previous studies have presumed that HPV E6 is the major survival determinant in human cervical cancer and this concept has directed anti-cancer pharmaceutical research towards identification of agents that block the functions of HPV E6. This has been a reasonable presumption given that HPV E6 targets the p53 tumour suppressor protein. However, our own previous [30] and present observations indicate that up-regulation of p53 following HPV E6 silencing is not sufficient to induce apoptosis in human cervical cancer cells despite induction of cell growth arrest.  HPV E7 silencing, on the other hand, induces apoptosis. Apoptosis of HPV E7-silenced SiHa cells proceeds despite the continued suppression of p53 by HPV E6 ([30,44] and Figure 5 schematic). The ability of HPV E7 to up-regulate SIRT1 expression now provides a mechanistic explanation for this effect since SIRT1 is anti-apoptotic in epithelial cancer cells (Figure 5).

Moreover, up-regulation of SIRT1 by HPV E7 also explains the attenuated functioning of p53 following selective HPV E6 depletion since the acetylation status of p53 under these conditions is low (Figure 4A; p53 K382Ac), consistent with its deacetylation by abnormally high levels of the SIRT1 de-acetylase (maintained by HPV E7; Figure 5). HPV E6 silencing nonetheless permitted up-regulation of the p53 target gene p21 ([30]; Figure 1D and 2D) and this would account for the cell growth arrest induced by selective silencing of HPV E6 in SiHa cells [30] (see Figure 5).

SIRT1 can function as a cancer-specific survival factor in cell lines derived from human epithelial and other cancers. Here we report that SIRT1 is targeted for up-regulation by the viral oncoprotein HPV E7. It is well established that HPV E6 and E7 function as co-operating onco-proteins and drive malignancy in cervical epithelial cells. Based on our present observations we suggest a new model for this co-operation (Figure 5) in which targeting of cellular p53 and SIRT1 by HPV E6 and E7 respectively enable dual access to the regulatory machinery normally involved in cellular homeostasis and chromosomal stability. In this way high risk HPV types subvert the SIRT1/p53 regulatory machinery from the process of aging to the process of cancerous malignancy.

## Materials and methods

Cell lines. SiHa and CaSki cell lines contain the HPV16 viral episome stably integrated into the host cell genome and were maintained and subcultured as described [30]. SiHa contain 1-2 integrated copies of the HPV16 episome per cell; CaSki have ~600 integrated copies per cell. Primary human keratinocytes were cultured in defined keratinocyte media (Gibco) with appropriate supplements.

siRNA transfection. Cells were transfected with HPLC-purified synthetic siRNAs (Qiagen) formulated into liposomes as described [20,30,45,46]. E6 and E7 siRNA sequences were as follows: E6 siRNA = 5'-GAGGUAUAUGACUUUGCUU(dTdT)-3'; E7 siRNA = 5'-AGGAGGAUGAAAUAGAUGG(dTdT)-3' as pub-lished [30]. E6b siRNA = 5'-GUUACCACAGUUAUG CACA(dTdT)-3'; E6c siRNA = 5'-AUCAUCAAGAAC ACGUAGA(dTdT)-3'; E7b siRNA = 5'-CAGAGCCCA UUACAAUAUU(dTdT)-3'. Other siRNA sequences were: survivin siRNA = 5'-GAGCCAAGAACAAAAU UGC(dTdT)-3'; aurora B siRNA = 5'-GGUGAUGGAG AAUAGCAGU(dTdT)-3', Itch siRNA = 5'-CCACAAC ACACGAAUUACA(dTdT)-3', c-FLIP siRNA = 5'-GCAGUCUGUUCAAGGAGCA(dTdT)-3'. Lamin A/C and SIRT1 siRNA sequences were as published [20]. Selectivity of the siRNAs and efficiency of silencing was confirmed as described previously [20,25,30,45,46].

E6 and E7 cDNA cloning and exogenous expression. Mammalian expression vectors for HPV16 E6 and HPV16 E7 were generated by subcloning HPV16 E6 cDNA or E7 cDNA, freshly cloned from low passage SiHa cells, into the multiple cloning site of pcDNA3.1. Generated constructs were verified by sequencing. DNA transfection was performed using lipofectamine (Invitrogen) according to the manufacturer's instructions as previously described [25].

mRNA quantification. Total cellular RNA was isolated using RNeasy kit (Qiagen) [46]. 100ng RNA was used for RT-PCR using Qiagen 1-Step RT-PCR kit or 50ng RNA for quantitative real-time RT-PCR on a DNA Engine Opticon2 system (MJ) using Quantitect SYBR Green RT-PCR kit (Qiagen). For survivin mRNA amplification primers 5'-GCATGGGTGCCCCGACGT TG-3' and 5'-TCAATCCATGGCAGCCAGCTG-3' were used in the thermal cycle: 50^0^C for 30min; 95^0^C for 15 min; 30 cycles of 94^0^C for 30 sec, 58^0^C for 45 sec, 72^0^C for 1 min; followed by 72^0^C for 5 min. For aurora B, primers 5'-ACAGACGGCTCCATCTGGCCT -3' and 5'-TCAGGCGACAGATTGAAGGGCA-3' were used in the thermal cycle: 50^0^C for 30min; 95^0^C for 15 min; 50 cycles of 94^0^C for 30 sec, 58^0^C for 45 sec, 72^0^C for 1 min; and then 72^0^C for 5 min. Cycle parameters and primers for HPV E6, HPV E7, SIRT1 and lamin A/C mRNAs were as previously described [20,30].

Immunoblotting. Total cell extracts were prepared from transfected cells [4] and equivalent amounts resolved by 15% SDS-PAGE and electroblotted onto nitrocellulose for immunoblotting. Antibodies used to detect specific post-translational histone modifications were: anti-phospho S10 H3 (Cell Signalling), anti-acetyl K18 H3 (Cell Signalling), anti-acetyl K9 H3 (Upstate Technology), anti-acetyl K14 H3 (Upstate), anti-dimethyl K9 H3 (Upstate) and anti-phospho S139 H2AX (Upstate). Other antibodies used were: anti-HPV16 E6 (ab70, Abcam), anti-HPV16 E7 (ED17, Santa Cruz), anti-p53 (DO1, Santa Cruz), anti-acetylated K382 p53 (Cell Signalling), anti-SIRT1 (Abcam), anti-S27P SIRT1 (Cell Signalling), anti-S47P  SIRT1 (Cell Signalling), anti-survivin (NB500-201, Novus Biologicals), anti-aurora B (BD Biosciences), anti-p21 (BD), anti-RB (G3-245, BD), anti-lamin A/C (636, Santa Cruz), and anti-actin (MAB1501, Chemicon). Actin was used as a loading reference control in all experiments.  Visualisation of bound antibody was by enhanced chemiluminescence (Roche). The intensity of bands was quantitated by densitometry using Quantity One software (Biorad) as previously [4]. Comparative analyses shown in Figure 2 employed data obtained from equivalent exposures with the selected antibody (eg. S10P, survivin). We thank Dr Jack Ford for the K382Ac antibody control (Figure 4A, bottom panel).

Cell cycle analysis and quantitation of apoptosis. Cell cycle analysis, and cell aggregate discrimination and histogram deconvolution using Cylchred software, were as previously described [4,20,45,46]. Apoptotic cells were identified by flow cytometry using Annexin V-Fluos (Roche) following the manufacturer's protocol [30].
